# Utility of ultrasound imaging in monitoring fracture healing in rat femur: Comparison with other imaging modalities

**DOI:** 10.1016/j.bonr.2024.101807

**Published:** 2024-09-23

**Authors:** Satoshi Inoue, Michinori Mori, Masaya Yasui, Miwako Matsuki-Fukushima, Kentaro Yoshimura, Naoko Nonaka

**Affiliations:** a Department of Oral Anatomy and Developmental Biology, Showa University School of Dentistry, 1-5-8 Hatanodai, Shinagawa-ku, Tokyo 142-8555, Japan; b Department of Judo Physical Therapy, Faculty of Health Care, Teikyo Heisei University, 2-51-4 Higashi-Ikebukuro, Toshimaku, Tokyo, Japan; c Department of Judo Seifuku and Health Sciences, Tokoha University, 1230 Miyakoda-Cho, Kita-Ku, Hamamatsu, Shizuoka 431-2102, Japan

**Keywords:** Ultrasound imaging, Radiography, Fracture healing process, Bony callus, Bone union

## Abstract

**Objective:**

Fractures are common injuries and various imaging modalities are employed to diagnose and monitor bone union. However, the follow-up of fracture healing using ultrasound imaging (US) remains a topic of debate. In this study, we analyzed of fracture healing process and compared US and radiological analyses with histological analyses to clarify the characteristics and limitations of each modality.

**Methods:**

An osteotomy model was created using the femur of Wistar rats, and US, radiological (radiography and micro-computed tomography (micro-CT)), and histological analyses were performed. Radiological assessments were conducted for the evaluation of calcified tissue. The gap between the bony callus and cartilaginous callus was measured.

**Results:**

US effectively captured changes on the fracture surface, potentially reflecting the early healing processes. Both US and radiographic findings showed strong correlation in terms of the decrease in the bony callus gap. US was unable to distinguish cartilaginous callus from the surrounding soft tissue. During the remodeling stage, micro-CT offered a detailed assessment of the internal fracture surface, whereas US was limited to evaluating the outer bone surface and lacked accuracy in visualizing the entire fracture site. Radiography provided a general overview of the fractures. The decrease in the bony callus gap measured using US correlated with the reduction in cartilaginous callus observed histologically.

**Conclusion:**

This study demonstrated that US could be a valuable tool for evaluating fracture healing. Combining fracture management with US and radiological examinations may provide a more accurate assessment of healing progress.

## Highlights

• Ultrasound effectively detected early changes in fracture surface during healing.
• Callus gap measurements by ultrasound correlated strongly with radiography.
• Combining ultrasound and radiography may improve fracture healing assessment.
• Ultrasound could be a valuable tool for evaluating fracture healing.

## 1 Introduction

Bone fractures occur at various anatomical sites (Driessen et al., 2016), and the fracture healing process is divided into four overlapping stages: inflammation, cartilaginous callus formation, bony callus formation, and remodeling (Salhotra et al., 2020). During this process, on the periosteal surface, a cartilaginous callus is formed around the fracture site, whereas a bony callus appears at the end of the fractured cortical bone. The cartilaginous callus is gradually replaced by bone through a process called endochondral ossification, ultimately leading to bridging and stabilization of the fracture.

Radiographs are the primary imaging modality used for the diagnosis of bone fractures (Parker and Johansen, 2006). There is a lack of consensus among orthopedic surgeons regarding the assessment of fracture healing, leading to differences in bone union evaluation time between surgeons (Nicholson et al., 2021). Bone union is also typically assessed using radiographs, with key indicators including callus bridging and fracture line disappearance (Corrales et al., 2008; Nicholson et al., 2021). These criteria are also important for predicting the progression of bone union (Nicholson et al., 2019). Bony callus detection on radiographs typically occurs 6–8 weeks after the injury (Cocco et al., 2022). Radiographs are two-dimensional projections and are thus prone to errors in judgment owing to overlapping bones and fixation devices. In addition, radiographs cannot be used to assess the crucial stages of inflammation and cartilage formation. One of the limitations of radiographs is its potential for radiation exposure and inability to effectively evaluate soft tissue and detect early-phase callus formation.

Imaging tools such as magnetic resonance imaging (MRI), computed tomography (CT), and ultrasound imaging (US) complement radiographs in diagnosing fractures, especially those that are difficult to detect, such as fatigue and insufficiency fractures (Cabarrus et al., 2008; Wright et al., 2016). CT can detect bony calluses prior to radiographs (Grigoryan et al., 2003), but the usefulness of MRI in bone union assessment remains unclear (Nicholson et al., 2021). Although MRI and CT offer superior visualization, high installation and maintenance costs and high patient burden restrict their routine use. CT has a higher radiation dose than radiography (Richardson, 2010), which is another factor that limits its use, especially in frequent follow-up examinations. Furthermore, both modalities require equipment installation in a clinical setting because of their high weight, which limits portability.

US is a promising alternative owing to its nonradiological, noninvasive, and real-time dynamic assessment of both bone and soft tissues (Moran and Thomson, 2020). Compared with other imaging modalities, US offers significant advantages in terms of portability and cost (Moran and Thomson, 2020). It is a valuable tool for the diagnosis of occult fractures, particularly in the ribs, and ankle fractures owing to its high resolution (Bianchi, 2020; Cocco et al., 2022). However, few studies have investigated the monitoring of the fracture healing process to bone union using US in humans and animals (Chachan et al., 2015; Chen et al., 2012; Maffulli and Thornton, 1995; Moed et al., 1998). In human tibial shaft fractures, bone union is diagnosed at an average of 9.74 weeks using US and 12.11 weeks using radiography, respectively (Chachan et al., 2015). These findings suggest that US may allow for earlier determination of bone union than radiography. In rabbit mandible healing, US and radiography findings show a correlation with the progression of bone union (Chen et al., 2012). However, the mandible differs from limb bones in its development, cellular composition, and healing process (Leucht et al., 2008). Thus, experiments using long bones are essential for understanding the healing process of extremity bone fractures.

Previous studies have focused on the relationship between US and radiological assessments, in fracture healing (Chachan et al., 2015; Chen et al., 2012; Maffulli and Thornton, 1995; Moed et al., 1998). However, no studies have investigated in detail how US reflects histological and radiological changes over time. In this study, radiological assessments were used for calcified tissue, and histological analysis was used for cartilage tissue. Radiography is used to visualize the entire fracture site, while micro-CT is used to observe its internal structure in detail. We compared US and radiological analyses (radiography and micro-CT), with histological analyses to clarify the characteristics and limitations of each modality.

## 2 Materials and methods

### 2.1 Animals

A total of 43 adult male Wistar rats weighing 253 (±9.4) g were purchased from Sankyo Laboratories (Tokyo, Japan). The rats were housed under specific pathogen-free conditions in a 12-h light-dark cycle and provided free access to food and water. The study was approved by the ethics committee of the Showa University School of Dentistry (approval number: 15012). Animal studies were performed in strict adherence to the guidelines and regulations for animal experimentation at our institution.

### 2.2 Operating procedure

**Fig. 1:**
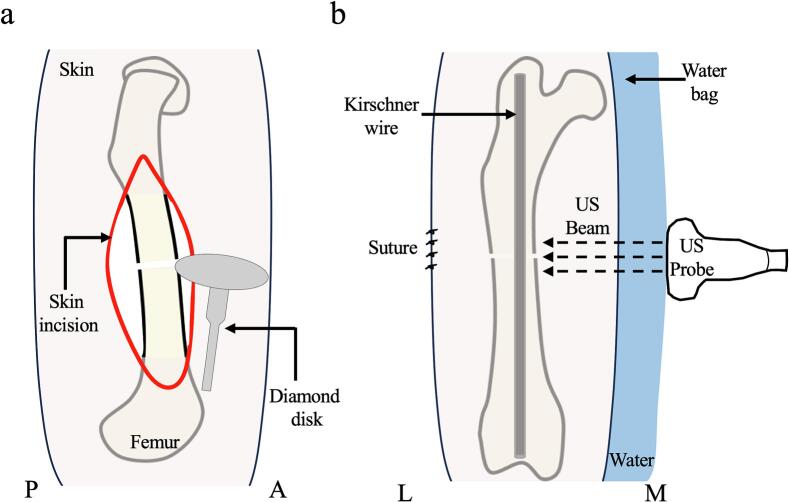
Fracture model and US observation a. Lateral view of femur. Osteotomy is performed from the lateral side to the medial side at the diaphysis of the femur. b. Anterior view of femur. US is performed from the medial side in the direction of the femoral long axis. US = ultrasound; A = anterior; P = posterior; L = lateral; M = medial.

**Fig. 2:**
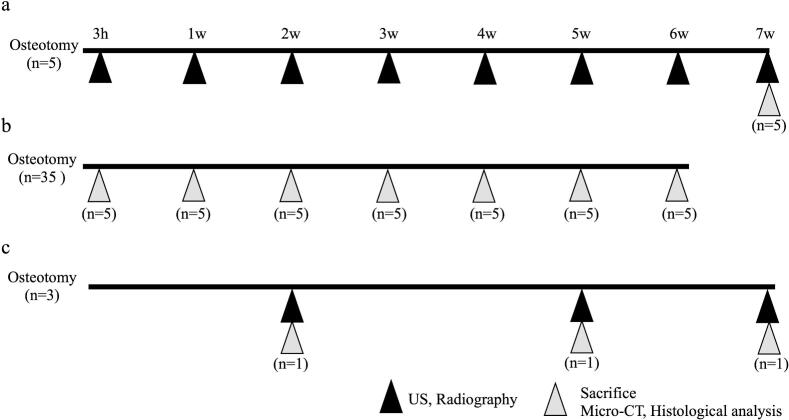
Experimental schedule a. The fractured femurs were observed using US and radiography from 3 h to 7 weeks. Rats were sacrificed at 7 weeks for micro-CT and histological analysis (*n* = 5). b. Fractured femurs were collected for micro-CT and histological analysis (n = 5/each time point). Micro-CT and histological analysis are performed after sacrifice. c. Representative imaging data were collected at 2, 5, and 7 weeks from individual rats (*n* = 1/each time point). All imaging modalities (US, radiography, micro-CT, and histological analysis) were performed at each time point. US = ultrasound; micro-CT = micro-computed tomography.

For osteotomy, the rats were anesthetized using a mixture of medetomidine (0.15 mg/kg), midazolam (2 mg/kg), and butorphanol (2.5 mg/kg). After shaving the lateral side of the right thigh, a longitudinal incision was made along the femoral shaft. The femur was exposed through the intermuscular space between the biceps femoris and vastus lateralis, and the periosteum of the diaphysis was incised longitudinally (Fig. 1a). An osteotomy was performed at the diaphysis of the femur using a diamond disk (diameter 1 mm, model #200 F10, Niigata Seiki, Niigata, Japan). The fractured femur was stabilized by the intramedullary insertion of a Kirschner wire (diameter 1 mm; MIZUHO, Tokyo, Japan) as described previously (Suen et al., 2014). The skin was sutured postoperatively. US and radiography were performed on the same rats (Fig. 2a). Micro-CT and histological analyses were performed on different rats (Fig. 2.b).

### 2.3 Photographs

Immediately after euthanasia with thiopental, the collected femurs were carefully excised from the surrounding soft tissue and photographed at each time point.

### 2.4 Ultrasound imaging

The fractured femur was observed using US at 3 h and weeks 1, 2, 3, 4, 5, 6, and 7 postoperatively (*n* = 5). To observe normal femoral structure, the unaffected side of the left femur was examined. US was performed using UGEO PT 60 A with a 30-MHz linear probe (Samsung Electronics Co., Seoul, Korea). A water bag filled with water was placed between the probe and the skin to ensure that the ultrasound beam entered the fracture site perpendicularly. The fractured site was observed from the medial side along the femoral long axis (Fig. 1b). The gap between the bony calluses was measured at the apex of both calluses using ImageJ software (NIH, Bethesda, MD, USA).

### 2.5 Radiography

Bone healing progress was assessed using radiographs obtained at 3 h and 1, 2, 3, 4, 5, 6, and 7 weeks postoperatively (n = 5). A radiography system (TRB 9020H, OBAYASHI MFG CO., Tokyo, Japan) was used with an exposure time of 60 s (90 mV, 2.5 mA). The direction of the radiographs was anterior to posterior and medial to lateral in the rat femurs. In some rats, the Kirschner wire was removed after euthanasia to observe the bony callus formation in detail. The bony callus formation on the medial side and the gap between the calluses was measured using ImageJ software. Measurement of the callus gap was performed using radiography and US, which are routinely used in clinical practice.

### 2.6 Micro-CT 3D images

Femurs from the fractured and non-fractured sides were fixed with 4 % paraformaldehyde in phosphate-buffered saline (PBS) at 4 °C. The fixed femurs were analyzed using ScanXmate-L090H (Comscan Techno, Kanagawa, Japan). To capture the entire callus formation area, the following imaging parameters were used 85 kV, 30 μA, voxel resolution 21 μm per voxel, and 992 × 992-pixel image matrices. Three-dimensional imaging data were reconstructed using the conneCT express software (White Rabbit, Tokyo, Japan). Bone mineral density BMD (mg/cm^3^) was measured in the periosteal callus using TRI-3D BON (Ratoc System Engineering Co., Tokyo, Japan). For BMD calibration, a phantom (Ratoc System Engineering Co., Tokyo, Japan) was scanned under the same conditions, and a calibration curve was created.

### 2.7 Histological analysis

After micro-CT analysis, femur samples were decalcified with formic acid‑sodium citrate for 1 week, embedded in paraffin, and sectioned into 5-mm thick sections. To confirm cartilaginous callus formation, sections were stained with hematoxylin and eosin (H&E) and safranin O/Fast Green. Stained images were obtained using a microscope (Bz-700-All-in-one; KEYENCE, Osaka, Japan). The safranin O-stained areas were identified based on their characteristic color, and their total areas were quantified (Bz-700-All-in-one; KEYENCE, Osaka, Japan).

### 2.8 Statistical analysis

The sample size was calculated (α = 0.05, power = 0.8) prior to the experiments using SPSS version 29 (SPSS, Chicago, IL, USA). Statistical analyses were performed using JMP® Pro 16 (SAS Institute Inc., Cary, NC, USA). Spearman's rank correlation coefficient was used to evaluate the relationships between US measurements of callus gap and radiographic findings, histological analysis of cartilaginous callus volume, and micro-CT measurements of bone mineral density (BMD) . A *p*-value <0.05 was considered statistically significant.

### 2.9 Representative imaging data

Data were collected at 2, 5, and 7 weeks when cartilage formation peaked, cortical bone continuity was restored, and bone union occurred. At these time points, we collected US, radiographs, micro-CT 3D, and histological images from the individual rats (Fig. 2c). The data collection was based on a statistical analysis of previous experiments, and only one representative example was presented (*n* = 1/each time point).

## 3 Results

### 3.1 Gross appearance of the fractured femur

**Fig. 3:**
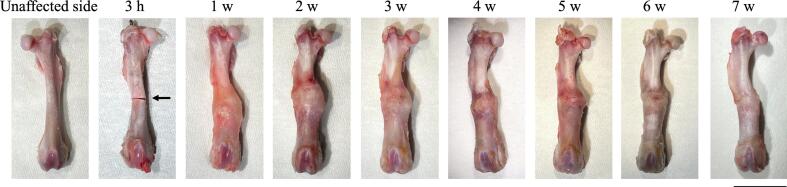
Gross appearance of the unaffected and fractured femurs Cortical continuity is observed on the unaffected side. The structure around the fracture site changes during the fracture healing process. Arrow indicates the fracture site. Bar = 1 cm.

After 3 h, the osteotomy divided the diaphysis of the right femur into two bone fragments, and the osteotomy site appeared linear (Fig. 3). After 1 week, loose connective tissue, which could not be exfoliated, formed and covered the osteotomy area in a spindle shape. From week 2 to week 3, the amount of coated connective tissue gradually increased, while the connective tissue altered detachable. From week 4 onwards, the overall size of the coated connective tissue gradually decreased.

### 3.2 Radiographic imaging

**Fig. 4:**
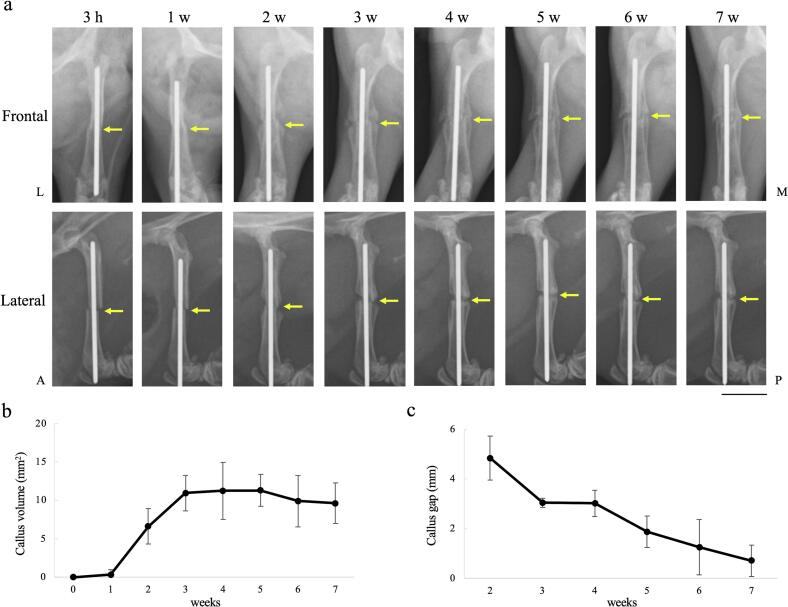
Representative radiographs during the fracture healing process (a) A radiographic images of the fracture healing process in the same rat. Bony callus formation is observed around the fracture site from week 2 onward, indicating progressive fracture repair. (b) Quantitative analysis of the callus volume and (c) the callus gap, presented as mean values (n = 5/ each time point). Arrows indicate the fracture site. L = lateral; M = medial; A = anterior; P = posterior. Bar = 1 cm.

Radiographs were taken from frontal and lateral angles. In contrast to the gross appearance, the soft connective tissue formed around the osteotomy was not depicted in the radiographs. Instead, the fractured femur and Kirschner wire were detected. The osteotomy site appeared consistent with the gross findings at 3 h. At week 1, no changes were observed around the osteotomy. At week 2, a bony callus had formed at the periphery of the fracture site on the periosteal side and increased until week 4 (Fig. 4a,b). The gap between the calluses gradually decreased until week 7 (Fig. 4c). At week 7, bone union was observed on the medial side, but not on the lateral side.

### 3.3 US imaging

**Fig. 5:**
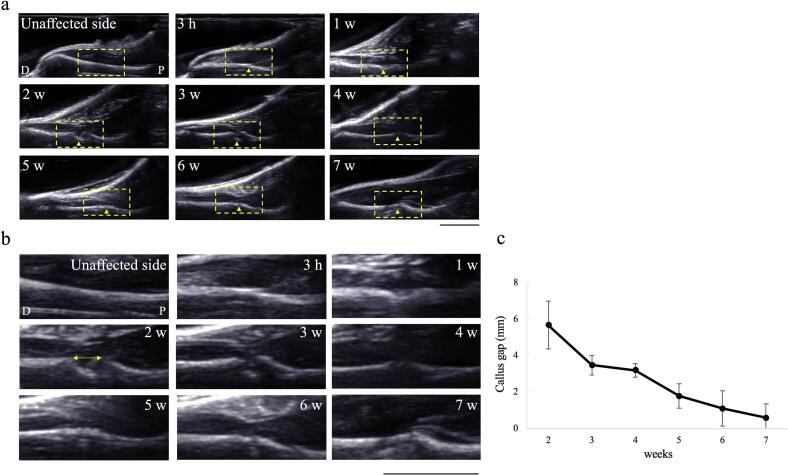
Representative US images during the fracture healing process (a) An US images of the fracture healing process in the same rat. The rectangular regions indicate the areas magnified in (b). (c) Quantitative analysis of the callus gap (n = 5/each time point). US is used to detect the detailed changes at the surface of the fracture site during the healing process. Arrowheads indicate the fracture site, and double-headed arrow indicate the callus gap. US = ultrasound. Bar = 1 cm.

US detected detailed changes on the surface of the fracture site. After 3 h, loss of continuity of the cortical bone was observed (Fig. 5a,b). At week 1, a slight elevation in the bony line was observed near the fracture site. At week 2, the bony line was further elevated, and a gap was formed at both sites. The gap between the calluses gradually decreased (Fig. 5c) and the continuity of the cortical bone was restored in some rats at week 5. Differentiation of cartilaginous callus formation was challenging using US.

### 3.4 Micro-CT 3D images

**Fig. 6:**
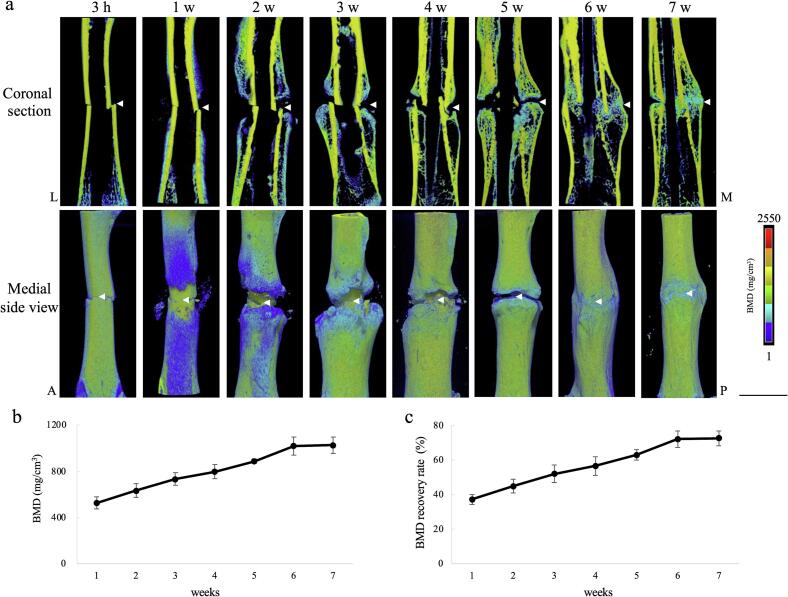
Micro-CT analysis of the fracture healing process (a) Micro-CT 3D images of coronal section and medial side view. Different rats are used for radiography (Fig. 4) and US (Fig. 5). The internal structure of the fracture site is observed in detail using micro-CT. (b) Quantitative analysis of BMD and (c) BMD recovery rate relative to the unaffected side (n = 5/each time point). Arrowheads indicate the fracture site. Micro-CT = micro-computed tomography; BMD = bone mineral density; L = lateral; M = medial; A = anterior; P = posterior. Bar = 5 mm.

Different experiment group of r rats were used for micro-CT and histological analyses using radiography and US (Fig. 2). At week 1, bony callus formation was observed near the fracture site (Fig. 6a). In week 2, **callus formation demonstrated further progress towards bridging the fracture.** The BMD of the bony callus gradually increased leading up to week 7 (Fig. 6b). Compared to the unaffected side, the BMD of the bony callus was approximately 45 %, 60 %, and 70 % at 2, 5, and 7 weeks, respectively (Fig. 6c).

### 3.5 Histological analysis

**Fig. 7:**
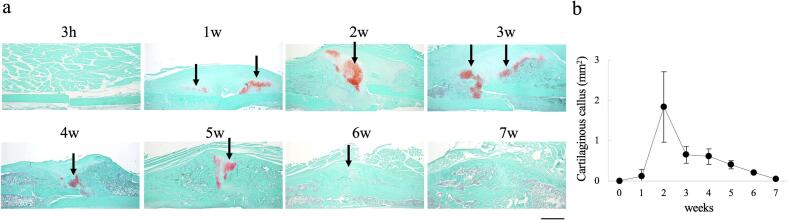
Cartilaginous callus formation during the bone repair process (a) Safranin O/fast green staining of the fractured femur. (b) Quantitative analysis of the area stained with safranin O (n = 5/each time point). Areas stained with Safranin O peak at 2 weeks. Arrows indicate stained with safranin O area. Bar = 1 mm.

Previous results indicated that cartilage formed during the healing process was not detectable by gross appearance observation or radiological examination and was difficult to identify using US. Therefore, demineralized paraffin specimens were prepared and subjected to safranin O/fast green staining to quantify the safranin O positive areas (Fig. 7a). After 1 week, safranin O staining was detected near the fracture site. Cartilage formation peaked at 2 weeks and gradually decreased thereafter (Fig. 7b). From week 2 onward, a cartilaginous callus was observed above the fracture site, located between the bony calluses.

### 3.6 Correlations between imaging modalities

**Fig. 8:**
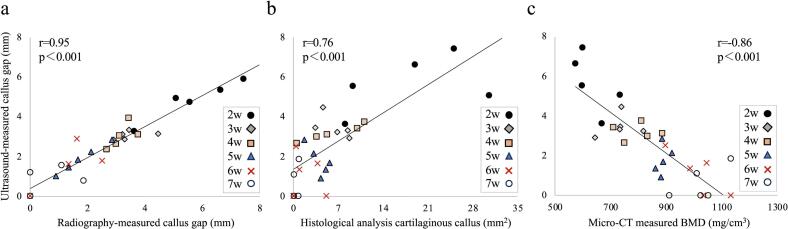
Correlation between US and other analyses Correlation between the callus gap measured using (a) US and radiography, (b) US and histological analyses, and (c) US and micro-CT (n = 5/each time point) . The US and radiography findings are consistent. In Figures a and b, some data points for weeks 6 and 7 overlap at value zero. US = ultrasound; micro-CT = micro-computed tomography.

A highly positive correlation was observed between the callus gap measured using US and radiography (*r* = 0.95, 95 % confidence interval (CL): 0.87 to 0.95), as well as between the amount of cartilage detected using histological analysis and the gap measured using US (*r* = 0.76, 95 % CL: 0.54 to 0.85) (Fig. 8a,b). A highly negative correlation was found between US measurements of the callus gap and micro-CT measurements of BMD (*r* = −0.86, 95 % CL: 0.63 to 0.90) (Fig. 8c).

### 3.7 Representative imaging data

**Fig. 9:**
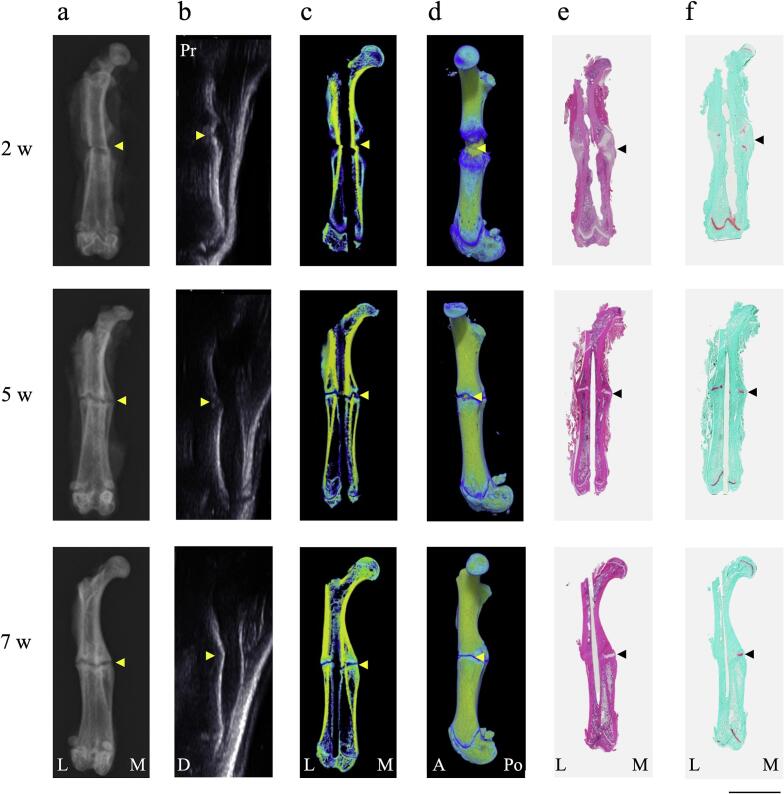
Representative imaging data (a) Radiographs. (b) US images. (c) Micro-CT 3D images of coronal section and (d) medial side view. Staining of the fractured femur with (e) hematoxylin and eosin and (f) Safranin O/fast green analysis. Image were analyzed at 2, 5, and 7 weeks (n = 1/each time point). The area stained with Safranin O in histological images is not differentiated using other modalities. Arrowheads indicate the fracture site. Pr = Proximal; D=Distal; L = lateral; M = medial; A = anterior; Po = posterior. Bar = 1 cm.

Radiographic, US, and histological analyses were performed on the individual rats at each time point (Fig. 9). The cartilaginous tissue observed in histological images was undetectable using other modalities. At week 5, bone union was observed on radiographs and US; however, micro-CT and histological analyses could not be observed. The gap between the calluses visible on radiological analysis was cartilaginous tissue. At week 7, bone union was observed, but the internal bone callus still contained cartilaginous tissue.

## 4 Discussion

This study investigated the ability of US to reflect histological and radiological changes over time in a rat fracture model. US detected changes in bony callus formation and progression, and a strong correlation was found between radiological and histological analyses. US was unable to distinguish cartilaginous callus from the surrounding soft tissue. Owing to its high water content, the fibrocartilage tissue exhibits an acoustic impedance similar to muscle and fat (Bhosale and Richardson, 2008; Shankar et al., 2011). This similarity presents difficulties for US in differentiating these tissues around a fracture site. Additionally, the smaller size of rats compared with humans further complicates the detection of cartilaginous tissue using US.

In the early stage of callus formation, bony callus formation was detected using US and micro-CT, but not radiography. Previous studies have reported that bony callus formation in humans is detected earlier with US (Nicholson et al., 2019) and micro-CT (Grigoryan et al., 2003) than with radiography. Our findings are consistent with these observations. In the early stages, the detection of bony callus formation using US reflects healing progression and is important for predicting non-union (Nicholson et al., 2021; Nicholson et al., 2019). These findings suggest that periodic US monitoring contributes to accurate evaluation of fracture healing in the early stages.

Each modality detects new bone formation during the bony callus formation stage. A previous US study utilized an invisible intramedullary nail from the fracture gap as a criterion for bone union (Moed et al., 1998). However, in certain cases, the US beam may be obstructed by cortical bone overlap at the fracture site, limiting the applicability of this method. Additionally, these criteria have not been used for conservative fracture treatments. Our study used callus-gap measurements to evaluate the progression of fracture healing. The decrease in callus gap on US correlated with that on radiography, a decrease in cartilaginous callus volume, and a negative correlation with increased BMD. These results suggest that callus gap measurement is useful for the evaluation of fracture healing progress.

During the remodeling stage, micro-CT offers a detailed assessment of the internal fracture surface, whereas US is limited to evaluating the outer bone surface and lacks accuracy in visualizing the entire fracture site, including the inferior aspect of the bony callus. Radiography provides a general overview of the fracture healing process but cannot offer the same level of detailed observation as CT.

Bone strength is a valuable indicator for guiding rehabilitation protocols and determining the optimal timing of fixation device removal. Bone strength at fracture site is estimated using radiography and CT. While accurate estimation of bone strength using US imaging remains challenging. US can be used to evaluate bone stability using dynamic stress tests, potentially providing an estimate of dynamic bone strength (Bianchi, 2020; Zhang et al., 2019). However, further research is needed to establish the relationship between US measurements and bone strength. In particular, when high-voltage radiological equipment is unavailable, such as in disaster situations, ultrasound-based estimation of bone strength becomes a necessary alternative.

The fracture healing process differs depending on the fracture site (Inoue et al., 2021; Inoue et al., 2020) and mechanical environment (Betts and Müller, 2014; Jagodzinski and Krettek, 2007). Fractures mainly occur in the metaphyseal regions of long bones such as the distal radius (Driessen et al., 2016; Hedström et al., 2010). In the metaphyseal region of long bones, fractures are repaired via intramembranous ossification from the side of the marrow cavity without periosteal callus formation (Inoue et al., 2021). US The fracture healing process in metaphyseal region may differ from this study. However, there are no reports on the effective evaluation of metaphyseal fractures using US. Future studies should focus on fracture healing in the absence of periosteal callus formation.

Our results suggest the possibility of US as an alternative radiological assessment tool for fracture union. In particular, US effectively captured changes on the fracture surface, potentially reflecting the early healing processes.

This study had several limitations. First, we initially estimated a sample size of 10 rats to achieve a correlation coefficient of 0.8. However, due to practical considerations, we reduced the sample size to 5 rats. As a result, the statistical power of this study is somewhat limited. Second, immunohistochemistry for inflammatory cells was not performed in histological analysis. Therefore, inflammatory cells could not be detected. Additionally, the hematoma that formed at the immediate fracture site could not be observed due to its loss during the process of preparing the paraffin specimens. Third, we utilized the osteotomy with intramedullary pin model for the fracture model. Hence, this study did not include the data of other types of fracture model such as closed fracture model, osteotomy with locking plate model, drill hole model or conservative treatment model. Further investigations are needed to determine whether similar findings can be replicated with different fracture models.

## 5 Conclusion

Our findings demonstrated that US could be a valuable tool for evaluating fracture healing. Combining fracture management with US and radiological examinations may provide a more accurate assessment of the healing progress.

## CRediT authorship contribution statement

**Satoshi Inoue:** Writing – review & editing, Writing – original draft, Project administration, Data curation, Conceptualization. **Michinori Mori:** Writing – review & editing, Methodology, Investigation, Data curation, Conceptualization. **Masaya Yasui:** Writing – review & editing, Methodology, Investigation, Data curation. **Miwako Matsuki-Fukushima:** Writing – review & editing, Data curation. **Kentaro Yoshimura:** Writing – review & editing, Data curation. **Naoko Nonaka:** Writing – review & editing, Supervision.

## Declaration of competing interest

The authors have no conflict of interest to declare.

## Data Availability

Data will be made available on request.
